# Exploring the experiences and strategies for promoting the well-being of first-time Black Canadian women transitioning to motherhood: A community-based participatory study protocol

**DOI:** 10.1177/17455057251385370

**Published:** 2025-10-22

**Authors:** Priscilla N. Boakye, Nadia Prendergast, Annette Bailey, Nana Ama Tiwaa-Boateng, Feven Desta, Mawuko Setordzi, Claire Zlobin

**Affiliations:** 1 Toronto Metropolitan University, Toronto, ON, Canada; 2 Western University, London, ON, Canada; 3 Life with a Baby, Thornhill, ON, Canada

**Keywords:** transition to motherhood, Black women, Canada, intersectionality, community cultural wealth

## Abstract

**Background:**

The arrival of a first child is often a transformative and joyful period filled with excitement, but it can also be an overwhelming and isolating experience, especially for first-time mothers facing structural and socio-economic disadvantages. Negative experiences during the early transition to motherhood may contribute to poor maternal mental health and affect the trajectory of infant development. Thus, supporting this journey is critical, yet there is a paucity of research on the experiences and support needs of first-time Black Canadian women transitioning to motherhood.

**Objective:**

The overall objective of this proposed study is to explore the experiences of transitioning to motherhood and to identify the contextual, sociocultural, and structural conditions that facilitate and or impede healthy transitions among first-time Black Canadian mothers.

**Design:**

This proposed study will employ a two-phase critical exploratory qualitative design guided by intersectionality, community cultural wealth, and a socio-ecological framework to explore the perspectives of first-time Black mothers on the contextual, cultural, and structural conditions that facilitate or impede healthy transition to motherhood.

**Methods:**

We will also draw on community-based participatory methods to engage them in translating the findings into accessible and actionable strategies to inform programs, interventions, and policies to support their transition needs. In collaboration with our community partner, six focus groups and 12 individual interviews will be conducted with first-time Black mothers from across Ontario. Data will be analysed using thematic analysis.

**Discussion:**

Most research on transition to motherhood has been conducted with white middle-class women. Understanding how Black mothers navigate their transition to motherhood through a complex array of entrenched systemic and racial inequities is critical to inform policy and development of interventions and programs to meet their transition needs including early screening of social and mental health risk factors.

**Conclusion:**

This project has the potential to inform social policy as it relates to parental leave, workplace accommodation, and accessible childcare to support first-time Black mothers transitioning to motherhood.

## Introduction

Becoming a first-time mother signals a shift into uncharted territory, which often evokes feelings of uncertainty. It is common for first-time mothers to be unprepared to take on the demands of motherhood, regardless of their age or culture.^1^ Thus, while first-time motherhood is often a transformative and joyful period, filled with excitement for some women,^2^ it can be an overwhelming and isolating experience for others, especially for first-time Black mothers facing structural and social-economic disadvantage.^3–5^ In the context of this study, we will be focusing on cisgender Black women who become mothers.

Transition to motherhood for Black mothers is often throttled by ongoing systemic and racial oppression, which may contribute to poor mental health and affect the trajectory of infant development. Research shows that promoting positive emotional well-being of first-time mothers is fundamental to their thriving and to improving the overall well-being of their children and families.^6^ Understanding how these outcomes are achieved for mothers whose experiences are modulated by structural oppression, is a salient gap in research.

Across many cultures, motherhood often is rooted in the concept of idealized motherhood, an assumption that women are expected to engage in an unconditional love and unwavering commitment of raising children irrespective of their life circumstances.^7^ As a result, motherhood is perceived as a natural occurrence for women,^1^ and thus, motherhood duties and responsibilities are declared the sole responsibility of women, an identity that is taken for granted across many cultures and contexts.^8,9^ Such beliefs about motherhood create pressures from unrealistic and unattainable expectations for women who are often employed outside of homes, while additionally assuming mothering responsibilities. These traditional expectations of motherhood are often inconsistent with the current contexts and realities of many women’s lives. However, expressing any struggles or challenges related to the pressures of aspiring to these standards of motherhood can further amplify experiences of dissatisfaction and discontentment among first-time mothers transitioning to motherhood.^2,9,10^

The transition to motherhood is associated with intense emotions and uncertainty, due to a constellation of physical, emotional, and psychological adjustments to maternal roles.^2,8^ A crisis of emotions related to isolation, despair, hatred, and sorrow, without support and preparation, can affect mothers’ emotional well-being and relationship with their child.^10^ For first-time Black mothers, historical and socio-political contexts may present barriers to accessing emotional support during this transition. While uncertainty, transformation, and reorganization are part of the transition to motherhood, the racial and economic context in which Black women are situated, in combination with beliefs and attitude towards motherhood, and awareness of and/or preparation for what to expect, differentiate this transition for them.^3,5,11^ Yet, there remains a lack of attention to the arduous aspects of this transition, particularly for Black mothers.

The intersections of race, class, and gender influence the social organization, the experience, and meaning assigned to motherhood.^11-14^ For example, the social organization of motherhood is associated with the ideology of intensive mothering – an expectation that assumes that mothers should invest all their material and emotional resources in caring for their children.^8,14,15^ This dominant view of motherhood has shaped the understanding and meaning of mothering, thereby, obscuring the inequalities, social, and material conditions in which motherhood takes place.^8,13,14,16^ The ideology of intensive mothering perpetuates a dualistic image of the ‘good’ and ‘bad’ mother, a standard against which mothers are evaluated.^8,10,17,18^ Mothers who conform with the ideal notions of motherhood (which are entangled with race and class) are deemed to be good and are often met with positive affirmations,^18^ those who do not align with the idealistic view of motherhood are often viewed with suspicion, perceived as deficient or bad, and are blamed, judged, and shamed.^3,4^ The ideal notion of the good mother as emotionally absorbing and the norm of intensive mothering reinforces motherhood as a locus of oppression with unrealistic expectations, especially for women from marginalized communities.^19,20^ Yet much transition to motherhood research has focused on the dysfunctional response of marginalized women to motherhood with very little attention paid to the specific social context of their life.

Navigating the transition to motherhood and adjusting to the demands of attending to infant care needs, engaging in multiple roles, financial strains, and transitioning back to work can feel profoundly overwhelming and stressful for first-time Black mothers, in a socially hostile context.^3,4,5^ Evidence from the United States suggests that 38% of first-time Black and racialized mothers experience unhealthy transitions to motherhood, characterized by poor mental health when compared with white women.^21^ Unhealthy transition refers to the difficulties women experience adapting to the physical, psycho-emotional, relational, and social changes associated with becoming mothers. Black mothers are more likely to experience higher rates of socio-economic disadvantage including living in poverty, being single, separated or divorced, unemployed, and facing financial distress.^3,4^ Combined with the stress of everyday racism and discrimination along with the lack of support, first-time Black mothers can feel intense pressure to succeed.^3,5,6^ When these intersecting challenges cumulate, they can devastate the emotional well-being of first-time Black mothers. The expectation to show strength, a characteristic expected of Black women and a demonstration of good mothering may contribute to first-time Black mothers’ unwillingness to openly discuss their emotional challenges, and their hesitancy to seek support.^13^ Without an outlet for relief or available social structures of support, many are left to rely on their internal capacities for perseverance and strength.^13^ This capacity to persevere to avoid being perceived as bad parent (often portrayed through the strong Black women trope) obscures their emotional needs and heightens their vulnerability to poor mental health outcomes.^19^

Unhealthy transitions and poor mental health among first-time mothers affect the quality of their relationship with their child, interfere with maternal role attainment, and undermine resilience and coping.^22^ Difficult transitions that interfere with a mother’s emotional response to infant needs and secure infant attachment can, in turn, affect the child’s social, emotional, and cognitive development and can lead to social and behavioural issues in later childhood.^23^ Thus, the impact of highly challenging motherhood transitions can persist throughout the developmental trajectory of children and may even lead to hostile parenting behaviours.^24^ Because work life is affected, poor transitions have also been found to exacerbate household socio-economic vulnerability, exposing children to poverty and increasing their risk to poorer health and longer-term developmental outcomes.^25^

The experiences of Black Canadian women transitioning to motherhood have not been extensively described in the literature, but it is likely that structural anti-Black racism and discrimination exacerbate many of the challenges described above. Much of the literature on transition to motherhood have focused on the idealistic perspective of motherhood and mainly conducted with white middle-class women, thus ignoring experiences of first-time Black mothers from an intersectional perspective and limiting the translation of the findings into meaningful practice and policy actions. Additionally, where motherhood is explored among Black populations, it tends to be deficit-focused, overlooking the protective assets and resources they use to navigate their transition to motherhood. Research shows that identifying the risks and strengthening protective factors such as internal coping resources, supportive relationships, and social networks are critical for promoting healthy transition to motherhood.^2^ Considering that more Black women delay childbearing due to education and career,^26^ and the limited research available on Black motherhood transitioning in Canada, it is critical to understand the diverse experiences of first-time Black Canadian mothers regardless of the age they become mothers.

## Research objectives

This proposed study aims to explore the experiences of first-time Black Canadian women transitioning to motherhood and to identify the contextual, sociocultural, and structural conditions that facilitate and or impede healthy transition. The specific objectives are:

- **RO1:** Explore first-time Black Canadian mothers’ experiences and perspectives on transition to motherhood.
- **RO2:** Identify the contextual, cultural, and structural conditions that influence their transition to motherhood.
- **RO3:** Identify the cultural assets and resources that support and influence their transition process.
- **RO4:** Engage them in translating the findings into accessible and actionable strategies to inform interventions and policies to support the transition needs of first-time Black Canadian mothers.

## Theoretical framework

This proposed study will be guided by Black feminist work on intersectionality,^27^ a community cultural wealth (CCW) framework,^28^ and socio-ecological framework (SEF).^29^ Intersectionality is rooted in the understanding that people experience discrimination and disadvantages at multiple levels and across multiple systems.^27^ It recognizes that people’s lives are shaped by multiple intersecting social identities and that the interaction between multiple social identities operates within an interlocking systems and structures of power.^27^ The transition to motherhood within the context of Black lives is uniquely shaped by the intersection of gender, race and class along with sociocultural norms and expectations. An intersectional lens takes into account how these intersecting identities and experiences shape the expectations and transition to motherhood. To understand the experiences of first-time Black mothers, intersectionality framework can help us clarify the ways that multiple systems of oppression work together to shape their experiences transitioning to motherhood.^27^

The CCW framework highlights the strategies and resources employed by marginalized groups to navigate systems of oppression.^28^ As a framework centred on community cultural assets/capital, it challenges the deficit-oriented approach that portrays marginalized groups as lacking the required capital and assets to thrive and succeed. Yosso^28^ identified six forms of capital including *aspirational, linguistic, familial, social, navigational, and resistance* that marginalized groups drawn on to succeed in the face of systemic barriers. Although Yosso’s theory provides a comprehensive framework for understanding the different forms of capital used by structurally marginalized people, however, not all aspect of the theory is relevant considering the focus of this research. Therefore, this, proposed study will focus on the last four forms of capital, that hold the most relevance to this inquiry –familial, social, navigational and resistance. Considering the focus of this study, *Familial capital* focuses on the strengths individuals draw from family knowledge, traditional norms and values, and family support to navigate their everyday life.^28^ This form of capital will help the research team explore and understand the role family, traditions, and knowledge play in first-time transition to motherhood. *Social capital* comprises community networks and resources that individuals from marginalized communities rely on to navigate and create pathways and opportunities, foster community resilience and pride, and promote collective empowerment.^28^ In the context of transition to motherhood, social capital will enable us to explore how community networks and resources influence the transition process.

*Navigational capital* consists of the skills and strategies used by communities to problem solve and thrive against oppressive systems and institutions.^28^ This form of capital will help us clarify how first-time mothers navigate different institutions such as health, childcare, and work as they transition to motherhood. *Resistance capital* refers to the knowledge, strategies, and skills used by marginalized communities to mobilize and confront different forms of oppression and injustice.^28^ This form of capital is intertwined with navigational capital and provides individuals with the consciousness needed to manoeuvre through and challenge systems of oppression that operate across multiple levels. Exploring resistance capital in the context of motherhood transition will help to uncover the ways first-time Black mothers challenge the sociocultural, political, and systemic inequalities that impact on their transition. CCW framework provides a powerful analytic tool that shifts attention away from deficits towards understanding how first-time Black mothers employ the different forms of capital as they transition to motherhood.

Combined with a SEF, through which we will analyse the complex interplay between individual, interpersonal, community, and societal level factors and their influence on transition to motherhood.^29^ Transition to motherhood is not only influenced by individual-level behaviours but instead is shaped by the interaction of complex multilevel systems. This framework considers how factors at the interpersonal, organizational, community, and societal level influences an individual’s behaviour, action, and outcome. SEF will allow us to understand and identify a range of factors and conditions including interpersonal, organizational policies, community, and societal level resources that shape the transition to motherhood. Through intersectionality, CCW, and SEF, we will explore conditions and factors at multiple levels to assess their influence on transition to motherhood and to identify areas for addressing obstacles, building on resources, and creating change.^29^ Bringing these frameworks together within Black feminist work helps to better situate Black mothers’ experiences of transitioning to motherhood and build understanding of Black mothers’ experience within the perspectives of oppression and exploitation.

## Overview of methods

**Figure 1.**
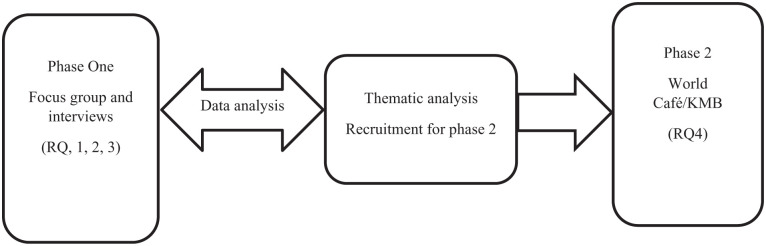
Project implementation process.

This proposed project will be conducted using a two-phase (Figure 1) critical exploratory qualitative design.^30^ Such a critical approach to inquiry aims to ‘expose and critique the forms of inequality and discrimination that operate in daily life’ (p. 9).^30^ We will draw on community-based participatory research (CBPR) methods to explore the contextual and structural influences on Black women as they transition to motherhood. This approach to research engages participants as active agents and co-creators of knowledge and takes into accounts diverse voices and perspectives to generate research output that is culturally and contextually grounded in the participants experiences ^31,32^ By actively involving participants in the research process, the findings of this current research project will offer critical insight into the contextual, sociocultural, and structural influences on transition to motherhood and contribute to identifying community-informed interventions, programs and policy recommendations needed to promote the well-being of Black women as they transition to motherhood.

## Sampling and study setting

The study will be conducted in Ontario, Canada and in collaboration with our community partner Life with a Baby. The inclusion criteria for participation are first-time mothers who: (1) self-identify as Black; (2) who have given birth in the last 1 year; and (3) be able to participate in English and reside in Ontario. We will recruit eligible first-time Black mothers through Life with a Baby, a community organization with eight local chapters across multiple locations in northern and southern Ontario. Each community chapter is managed by a Community Chapter Manager and multiple volunteers who work with new mothers to understand their needs and help connect them with resources. The organization also maintains an active online chapter to provide virtual support for new mothers without a local chapter. With the guidance of local chapters, we will also outreach to a wider network of community stakeholders including Black mothers’ groups, faith-based organizations, Black businesses, salons, Black churches, community, and neighbourhood associations in each of the eight regions as needed. We will foster meaningful community engagement with Life with a Baby, which will help us ensure that our findings and knowledge mobilization (KMb) activities are locally relevant. Data collection and analysis for all phases will take place between July 2025 and June 2026.

## Phase one (RO 1, 2, and 3): exploring perspectives and contextual influences

In this phase, we will use focus groups and in-depth interviews to explore the perspectives and contextual influences on Black mothers’ transition to motherhood. These methods will enable us to explore and triangulate across multiple intersectional factors, cultural assets and community and society-level factors to better understand their impacts on the transition process.

## Focus groups

As little is known about the transition to motherhood among Black populations, we will begin data collection with focus groups,^33^ and aim to recruit a diverse sample from across Ontario. We will hold 6 focus groups with a maximum of 8 participants each (*N* = 48); this is a large enough sample to allow for diversity in location, access to resources, sociodemographic backgrounds, and understandings of motherhood. Furthermore, given the scope of this study, a sample of 48 for the focus group is deemed adequate to allow for sharing of diverse experiences and perspectives as well as enable the capturing of rich and contextual understanding of the individual and shared meaning. Limiting the focus groups to eight will allow for equal participation and encourage discussion. This sample will not be representative of the entire population of first-time Black mothers in Canada, but it will capture the heterogeneity of this population in Ontario. We are confident in our ability to recruit this number of participants based on our estimates of the population of first-time Black mothers in Ontario 1 year following childbirth.^34^ Six focus groups should allow us to achieve code and meaning saturation with the level of heterogeneity we anticipate in our sample.^35^ Focus groups will be held virtually in order to facilitate participation from across Ontario. We will create opportunity for in-person participation for those who may have issues with accessing technology and participants will be reimbursed for transportation. The focus groups will be led by the research team who will facilitate opportunities for all participants to share and exchange information, comparing experiences across geographic and social locations, leading to a deeper, comprehensive, and collective understanding. To enhance access to equitable participation, we will identify and direct questions to those who may not be engaging in the discussion. Additionally, we will ask participants to use the private chat for sensitive information they may feel uncomfortable to share within the group. We will also create opportunity for one in-person focus for those who may be experiencing barriers related to technology. Data analysis will be ongoing with emergent themes explored in subsequent focus groups. At the end of each focus group, participants will be asked to indicate if they would like to be invited for follow-up interview and or the World Café session.

## Individual interviews

Following preliminary analysis of the focus group data, we will purposely select from the pool of focus group participants who express interest in joining the in-depth follow-up interviews. The selection of participants will be guided by a purposive sampling matrix^36^ to ensure we attain maximum diversity based on the range of experiences, backgrounds, and demographic profiles, and expected outcome of the study. A total of 12 participants will be invited for the interview. These interviews will allow us to follow-up on important themes identified in the focus groups and to provide participants with an opportunity to share details that they may not have felt comfortable sharing in a group setting. Both interviews and focus groups will be audio-recorded.

## Data analysis and interpretation

The audio recording from the focus groups and individual interviews will be transcribed into text. We will engage graduate research assistants to ensure the audios are accurately transcribed. Additionally, senior members of the research team will review the audio and transcript and assess the accuracy of the transcription. The transcripts will be de-identified of all personal information and exported to NVivo for organization and management of the analytic process. Consistent with qualitative research, data analysis will be done concurrently with data collection to allow the research team to probe emerging themes with subsequent focus groups and interviews.

A thematic analysis will be informed by Braun and Clarke^37^ six stage approach to facilitate individual and collective insights on Black women’s transition to motherhood. Thematic analysis is not linear but instead an iterative process that allows researchers to move back and forth between the different stages of analysis.^37^ The thematic analysis will be guided by the following steps: (1) reading and familiarizing with the data, (2) generating the initial codes and applying the codes to the data, (3) sorting of the data and generating themes, (4) reviewing of tentative themes, (5) naming and defining the themes and their relationship, and (6) writing a report to capture the data interpretation and essence of each theme.^39^ Drawing on the theoretical frameworks guiding this study, we will conduct thematic analysis using inductive and deductive techniques.^37^ The data will first be analysed inductively to understand the nuances in the experiences of the participant and subsequently we will draw on the theoretical framework to inform the deductive analysis. We will draw on intersectionality framework to explore ways race, gender, class and other identities influence and shape the journeys of first-time Black mothers transitioning to motherhood. Alongside, we will apply CCW to analyse the different forms of cultural capital that Black mothers employ to navigate the structural conditions that influence their transition process. To ensure consistency with our coding framework and enhance the trustworthiness of our findings, we two members of the research team will conduct the data analysis, and all team members will meet frequently to discuss the process and build consensus.

## Phase 2 (RO 4): Integrated KMb engagements and stakeholder forum

CBPR emphasizes the importance of engaging participants to collectively translate the findings of a research into accessible and actionable strategies that are meaningful to community.^32^ This will ensure participants can directly inform and influence policies, programmes, and interventions that addressed their needs. We will, therefore, use principles of meaningful community engagement to actively engage participants as partners in knowledge co-creation. We will use the World Café Method as a participatory tool to facilitate knowledge translation (KMb) and to identify required areas for change.^38^ As a participatory method, World Café is an effective strategy for knowledge co-creation, facilitating equitable participation and engaging participants to collectively translate the evidence into actionable strategies.^39,40^ We will engage 30 participants from Phase 1 who expressed interest in participating in this phase of the project. We will share with them the findings of the focus groups and interviews along with synthesis from existing literature, and they will be invited to reflect on this knowledge to discuss what needs to be done by various stakeholders to support the needs of Black mothers’ transitioning to motherhood.

Participants will be asked to work in small groups to generate ideas and strategies. Each group will be facilitated by the research team members, and each group will share their insights from their discussion with the larger group. This process allows for cross-pollination and connection of diverse perspectives and ideas to facilitate shared collective discoveries.^40^ Guided by social ecological framework, the team will work with participants to cluster and arrange their responses according to individual, interpersonal, community, organizational and societal level using pictorial format. We will also record, transcribe, and analyse the data generated to inform the development of KMb packages including policy briefs and public-facing infographics. We will translate the ideas and expressions into Shespeaks digital stories to communicate Black mothers lived experiences in empowering ways and to connect with wider audiences and stakeholders. The Shepeaks digital stories will be available in video and picture format to ensure wider accessibility. Additionally, the videos will be available in YouTube format, and the link will be shared via the website of our community partner Life with a Baby and their affiliate organizations. Using this approach, we will create an empowering and effective environment for authentic engagement with first-time Black mothers.

We will also organize an intersectoral multi-stakeholder KMb forum to take place in the final month of the grant period through which we will promote knowledge exchange and uptake; and facilitate the establishment of collaborative networks. We will engage 40-key stakeholders from across Ontario: decision makers, parent advocates/champions, faith-based organizations and churches, community organizations and associations, parent navigator groups, and Black community groups to raise awareness and promote partnership and networking to advance policy. We will work with our community partner Life with a Baby to select participants for the KMb forum to ensure representation of relevant stakeholders including policy makers, knowledge users, and parent advocacy groups. It is anticipated that the end of grant forum will highlight priority areas for policy action to address the structural issues impacting on healthy transition to motherhood and strengthen protective factors with the existing family-centred maternity and newborn care national guidelines.

## Expected outcomes

Through our comprehensive KMb plan, public, policy, and practitioner, audiences will understand the emotional needs and well-being of first-time Black mothers. Not only will we uncover missing supports and challenges, but our study will also highlight the protective assets of Black mothers to expose and build on their ability to flourish and thrive. This knowledge is critically needed to inform the integration of these protective assets in the development of practice, program, and policy interventions to support the emotional well-being of Black mothers as they transition to motherhood. The findings of this study will raise public awareness of their struggles as well as their resiliencies, which can help break down harmful stereotypes and tropes whether they stigmatize Black mothers or hold them up to unattainably high expectations. Via our KMb activities and collaborator’s involvement, our findings will inform social care policies and the development of culturally meaningful and community-centred programs to promote the well-being of first-time Black mothers and their infants in Ontario and beyond.

## Trustworthiness

The strategies for evaluating the trustworthiness of this research will be based on the synthesis of techniques as they relate to credibility, transferability, dependability, and confirmability. Strategies for achieving credibility in this research will include researcher and data triangulation using different data collection methods (focus group, interview, and World Café), participant validation, debriefing, and reflexive journaling.^41^ To achieve validation, we will ask participants to clarify statements, and the research team will probe for further details during the focus group and interview to ensure accurate understanding. Using different data sources will contribute to adding depth and scope to the research findings and provide multiple views of the phenomenon under study. We will keep a reflexive journal of our own perceptions, biases, beliefs and thoughts and how these factors may influence the analysis and interpretation of the data. We will facilitate the transferability of our findings by providing sufficient contextual information about our participants and the research setting. In this study, we will achieve dependability by keeping an audit trail of the analysis and interpretation. We will establish confirmability through an audit trail and reflexivity. The research team will acknowledge and document our social location as Black scholars as it relates to our personal feelings, preconceptions, and contextual influences that shape our experience and those of our participants.

## Expertise of the research team

The team consists of researchers with relevant and complementary expertise and experience working with Black mothers, families, and communities. Our research programs focus on promoting well-being, flourishing, and advancing equity among Black populations including mothers. The team members have expertise and experience in community-based research, advocacy, project planning, and qualitative inquiry. The team will work closely with our collaborator to coordinate the recruitment of participants. Together the team brings the requisite research skills, content expertise, and KMb experience to successfully implement the project and proposed activities.

## Discussion

This project will generate much-needed empirical knowledge on the experiences, needs, and conditions that promote or impede the well-being of first-time Black mothers. Much of what is known about first-time Black mothers comes from the American context and mostly deficit-oriented. Motherhood transition research tends to focus on white, middle-class women; our theoretical lens and focus on Black women will contribute new insights into the social construction of this transition. Our methods will also offer insights into inclusive research designs and meaningful engagement with first-time Black mothers. Findings can inform more comprehensive long-term research in this highly under-explored area.

## Strengths and limitations

The use of a community-based participatory approach, and our integrated KMb strategies to gain in-depth understanding and to identify policy and priority areas for action is a key strength of this study. We anticipate that collecting the data from the province of Ontario might limit the applicability of the findings to other Canadian contexts. However, given that more than half of Black populations in Canada live in Ontario and that structural racism may impact on transition to motherhood in similar ways, the findings will provide meaningful direction for advancing equity for first-time Black mothers.

## Conclusion

This proposed study will shift the dominant ways of understanding transition to motherhood toward generating knowledge on contextual influences on Black women’s transition and the different forms of capital and strategies they use to navigate this transition process. The findings will highlight critical priority areas to inform social policy and guide the development of interventions and programs to promote healthy transition to motherhood.
